# Treatment strategies for high resveratrol induction in *Vitis vinifera* L. cell suspension culture

**DOI:** 10.1016/j.btre.2014.04.002

**Published:** 2014-05-19

**Authors:** Thu V. Vuong, Chris Franco, Wei Zhang

**Affiliations:** a Department of Medical Biotechnology, School of Medicine, Flinders University, Adelaide 5042, Australia; b Department of Chemical Engineering and Applied Chemistry, University of Toronto, Toronto, Ontario, Canada

**Keywords:** *Vitis vinifera*, Cell suspension culture, Resveratrol, Stilbene, Elicitation, *In situ* adsorption

## Abstract

Bioprocesses capable of producing large scales of resveratrol at nutraceutical grade are in demand. This study herein investigated treatment strategies to induce the production of resveratrol in *Vitis vinifera* L. cell suspension cultures. Among seven investigated elicitors, jasmonic acid (JA), salicylic acid, β-glucan (GLU), and chitosan enhanced the production of intracellular resveratrol manyfold. The combined treatment of JA and GLU increased extracellular resveratrol production by up to tenfold. The application of Amberlite XAD-7 resin for *in situ* removal and artificial storage of secreted resveratrol further increased resveratrol production by up to four orders of magnitude. The level of resveratrol produced in response to the combined treatment with 200 g/L XAD-7, 10 μM JA and 1 mg/mL GLU was approximately 2400 mg/L, allowing the production of resveratrol at an industrial scale. The high yield of resveratrol is due to the involvement of a number of mechanisms working in concert.

## 1 Introduction

One of the most widespread stilbenes is resveratrol (C_14_H_12_O_3_). The biosynthesis of resveratrol is controlled by stilbene synthase (EC 2.3.1.95). This enzyme uses the same substrates and catalyzes the same condensing-type enzyme reaction as chalcone synthase (EC. 2.3.1.74), which is involved in the biosynthesis of flavonoids including anthocyanins 1, 2. Resveratrol is then the skeleton for producing other stilbenes; for instance, a glycosylation of resveratrol can lead to piceid while an oxidative dimerization of resveratrol units can form ɛ-viniferin, a resveratrol dehydrodimer [3]. Resveratrol has been found to have a number of health benefits: Bradamante et al. [4] revealed that resveratrol prevented heart-artery diseases by reducing cholesterol and harmful blot clots, and hardening of the arteries. Resveratrol also showed cancer chemopreventive and therapeutic effects [5], and it can act as a neuroprotectant [6].

Due to these health benefits, there is an increasing demand for effective approaches to produce resveratrol. Although this compound can be chemically synthesized [7], the need for a safe and green product is in favor of using natural sources. However, the production of resveratrol directly from plants confronts a number of drawbacks, such as yield variation, pathogens, low purity and a long growth period. Thus, plant cell culture is preferred as this approach overcomes those obstacles while ensuring a continuous supply of products in uniform quality [8], which is important to industrial bioprocesses. Grape cell suspension cultures have been reported to accumulate stilbenes including *trans*-resveratrol, *trans*/*cis*-piceid, ɛ-viniferin, δ-viniferin, pterostilbene, and *trans*-astringin 9, 10. However, the accumulation of resveratrol in untreated grape cell cultures is low, less than 0.01% of dry weight or 2–3 mg/L [11].

The production of secondary metabolites in plant cell and tissue cultures can be enhanced by elicitors [12]. A number of elicitors including UV, methyl jasmonate, and indanoyl-isoleucine triggered the production of secondary metabolites, including resveratrol 10, 13, 14, 15, 16; however, the roles of many other potential elicitors remain to be investigated. If secondary metabolites are secreted, *in situ* adsorption is considered. Amberlite XAD-7, hereafter XAD-7, surpassed other XAD adsorbents in adsorption of many antioxidants including α-tocopherol and α-tocopheryl acetate, which share several common characteristics of resveratrol [17]. *In situ* adsorption might be crucial, as exogenous resveratrol at a concentration greater than 100 μM or 22.8 mg/L inhibited cell growth of *V. vinifera* cv. ‘Pinot Noir’ in a dose-dependent manner [18].

In this study, the elicitation of seven compounds, including jasmonic acid (JA), salicylic acid (SA), 3-methyl-salicyclic acid (MeSA), betaine (BET), β-glucan (GLU), methyl-β-cyclodextrin (MeCD) and chitosan (CHI) was investigated in single and combined treatments for enhancing the production of resveratrol in *V. vinifera* L. cv. Gamay Fréaux cell suspension cultures. As resveratrol was found secreted into the medium, the elicitation technology was then combined with *in situ* adsorption and artificial extracellular storage for optimizing resveratrol production, with a view toward large-scale production.

## 2 Materials and methods

Unless indicated, all chemicals were purchased from Sigma (Australia).

### 2.1 Cell suspension culture

The *V. vinifera* L. cv. Gamay Fréaux cell line was a gift from Dr. Francois Cormier (Québec, Canada). This cell line was grown in GC-2 medium pH 5.7–5.8, which is B5 medium supplemented with 30 g/L sucrose, 250 mg/L casein hydrolysate, 0.1 mg/L α-naphthaleneacetic acid, and 0.2 mg/L kinetin. Cell suspension cultures were maintained on a reciprocating shaker (Ratek Instruments, Australia) at 100 strokes/min at 27 ± 1 °C. The cultures were kept in the dark to prevent the biosynthesis of anthocyanins that complete with resveratrol and other stilbenes for the same precursors.

### 2.2 Elicitor treatment procedure

Pre-cultured 7-day-old cell suspensions were filtered through a 50 μm stainless mesh (Endecotts Ltd. London, England), and the cells were transferred in 20 mL fresh GC-2 medium to reach the concentration of 50 g fresh cells/L. The flasks in triplicate were incubated on a reciprocal shaker (Ratek Instruments, Australia) at 100 strokes/min in a dark, temperature-controlled room at 27 ± 1 °C. Elicitors were added after 4 days of culture when cells begin their log phase of growth [19].

The stock solutions of JA, SA, MeSA (Aldrich, Australia), MeCD (Aldrich, Australia), CHI, BET and GLU were filter-sterilized through a 0.22 μm Millipore filter (Minisart^®^, Sartorius, Germany). Less than 50 μL of elicitor solutions (or 1/400 of the final culture volume) were added to avoid any adverse effects of the solvents. Samples in triplicate were taken on day 4, and on every three days after the addition of elicitors.

### 2.3 XAD-7 pre-treatment

XAD-7 with an average pore diameter of 90 Å and surface area of 450 m^2^/g was used. XAD-7 beads were first soaked in 100% methanol for 30 min at room temperature (RT). They were then washed 3 times with MilliQ water on a filter unit with Whatman#1 filter paper (Whatman International Ltd., England) to remove traces of methanol, and left at RT to dry. XAD-7 beads were weighed and placed (20 g/L and 200 g/L XAD-7) in each flask before the medium GC-2 was added.

### 2.4 Elicitation and sampling in XAD-7 experiments

Ten mL GC-2 containing 1 g of fresh cells was transferred to 100 mL Erlenmeyer flasks containing 10 mL medium with the desired concentration of XAD-7. Thus, cells were grown with XAD-7 before the treatment of elicitors. At every sampling point, mixture of cells and XAD-7 from each flask was centrifuged at 2500 × *g* for 5 min at 4 °C using an IEC Centra-8R centrifuge (International Equipment Company, USA). Then, 200 μL medium from each tube was taken for the total extracellular phenolics analysis and 10 mL medium was for the analysis of extracellular stilbene. The cell and bead samples were filtered through a Whatman#1 filter paper (Whatman International Ltd., England) and dried in the oven for dry weight measurements.

For extraction of stilbenes from XAD-7 beads, samples were transferred into 20% sucrose solution, and gently stirred at the liquid surface to promote bead separation. Grape cells, which remain suspended, were removed by pipetting and the settled bead phase was vacuum filtered. Dried beads were weighed and then extracted for 1 h in 100% methanol with a volume equivalent to 20-fold of bead weight. The liquid phase was collected for HPLC analysis. All procedures were conducted in dim light to avoid photochemical alterations of stilbenes.

### 2.5 Cell growth measurement

During a culture cycle, approximately 2–3 mL volume of cell suspension from each flask was taken and centrifuged at 2000 × *g* for 5 min at 4 °C (IEC Centra-8R centrifuge, USA). The fresh cells were taken and weighed on pieces of aluminum foil, which were pre-dried at least 30 min in 70–80 °C oven. The remaining cells were dried for 2 days in a 70–80 °C oven to calculate the dry cell weight (DCW).

### 2.6 Phenolics extraction and measurement

The phenolics concentrations were measured using a modification of the Folin–Ciocalteu technique described by Singleton and Rossi [20]. About 40 mg of fresh cells was homogenized in a 20-fold volume of 100% ethanol (Merck, Australia) containing 0.1% HCl for 1 min at 22100–24500 rpm by using a homogenizer (CAT X120, Germany). The homogenate was left for 30 min at RT for extraction. After being centrifuged for 10 min at 7500 **×** *g* (Mikro 12-24, Hettich, Germany), the supernatant was collected. The supernatant (for intracellular phenolics) or the medium (for extracellular phenolics) were added with a sufficient amount of the Folin–Ciocalteu reagent, vortexed and incubated for 7 min at RT. The chemical reaction was terminated by 20% sodium carbonate solution (Aldrich, Australia). The absorbance at 760 nm was measured in a UV mini-1240 spectrophotometer (Shimadzu, Japan) to calculate the concentration of phenolics, using gallic acid (3,4,5-trihydroxybenzoic acid) as the standard.

### 2.7 Anthocyanin extraction and measurement

Procedures were carried out in dim light as a portion of extract was also used for stilbene analysis. Fifty to sixty mg of fresh cells was extracted with an acidified methanol solution (0.1% HCl) of 20-fold volume equivalent to the fresh cell weight. The resultant suspension was vortexed and incubated overnight at 4 °C for a complete extraction, and then microcentrifuged at 12000 × *g* for 5 min (Mikro 12-24, Hettich, Germany). A portion of the supernatant was measured at *A*_530 nm_ (UV mini-1240, Shimadzu, Japan) for quantification of anthocyanins using cyanidin-3-monoglucoside, one of the major anthocyanins in *V. vinifera* L. grape extracts [21], as the standard. The remaining supernatant was for analysis of stilbenes by HPLC.

### 2.8 Extracellular stilbene extraction

The culture was centrifuged at 2500 × *g* for 10 min at 4 °C in an IEC Centra-8R centrifuge (International Equipment Company, USA). 10 mL of the supernatant was added to 10 mL of 100% ethyl acetate (Aldrich, Australia), and mixed thoroughly for 5 min. The mixture was left at RT for 30 min to allow phases to settle, and then the upper phase was collected. The extraction was repeated to completely extract all the stilbenes in the medium. The upper phase was vacuum dried in a concentrator system (Centrivap, Labconco, USA) for around 3 h until all the ethyl acetate was evaporated. The pellet was resuspended in 100 μL 100% methanol, and kept at −20 °C for HPLC analysis. All procedures were conducted in dim light.

### 2.9 Stilbene determination

Stilbene samples were analyzed by an HPLC system (Shimadzu LC-10ADVP, Japan), which consisted of a HPLC pump LC-10 ADVP, a system controller SCL-10AVP, an autoinjector SIL-10ADVP, an on-line degasser DGU14A, a multisolvent selector FCV-10ALvp and a UV/VIS photodiode array detector SPD-M10AVP. Prior to HPLC analysis, all extracts were centrifuged at 15000 × *g* for 15 min (Mikro 12-24, Hettich, Germany). Reversed-phase chromatographic separation was conducted on an Apollo 5 μm C18, 250 mm × 4.6 mm-internal diameter column (Alltech, Australia). Elution was performed with a linear gradient of 0–95% HPLC-grade acetonitrile (Riedel-de Haën, Germany) in 20% acetonitrile for 35 min with the flow rate of 1 mL/min. The eluent was monitored at 307 nm and 285 nm, which are the maximum UV absorbancies of *trans*- and *cis*-resveratrol respectively [9]. *Trans*-resveratrol and *trans*-piceid standards were obtained from Polyphenols (Sandnes, Norway).

## 3 Results and discussion

The effects of elicitation were evaluated on cell growth as well as on the production of phenolics, anthocyanins and stilbenes.

### 3.1 *V. vinifera* L. cell growth

Among single elicitation treatments, SA at a concentration of 500 μM and MeSA at concentrations greater than 300 μM, besides GLU, decreased cell growth. In the treatment with 500 μM SA and 600 μM MeSA, the dry cell weight (DCW) at day 10 decreased by approximately 30%, when compared with the control (Table 1). The DCW decrease by GLU did not significantly affect the total intracellular phenolics. Whereas, SA and MeSA at those high concentrations dramatically reduced the intracellular phenolics while increasing the extracellular counterpart (Table 1), indicating the release of phenolics components, probably due to broken cells.

### 3.2 Production of intracellular resveratrol and anthocyanins

As anthocyanins are stored in vacuoles, and their biosynthesis is related to that of resveratrol, the intracellular production of these secondary metabolites was evaluated at the same time.

**Fig. 1:**
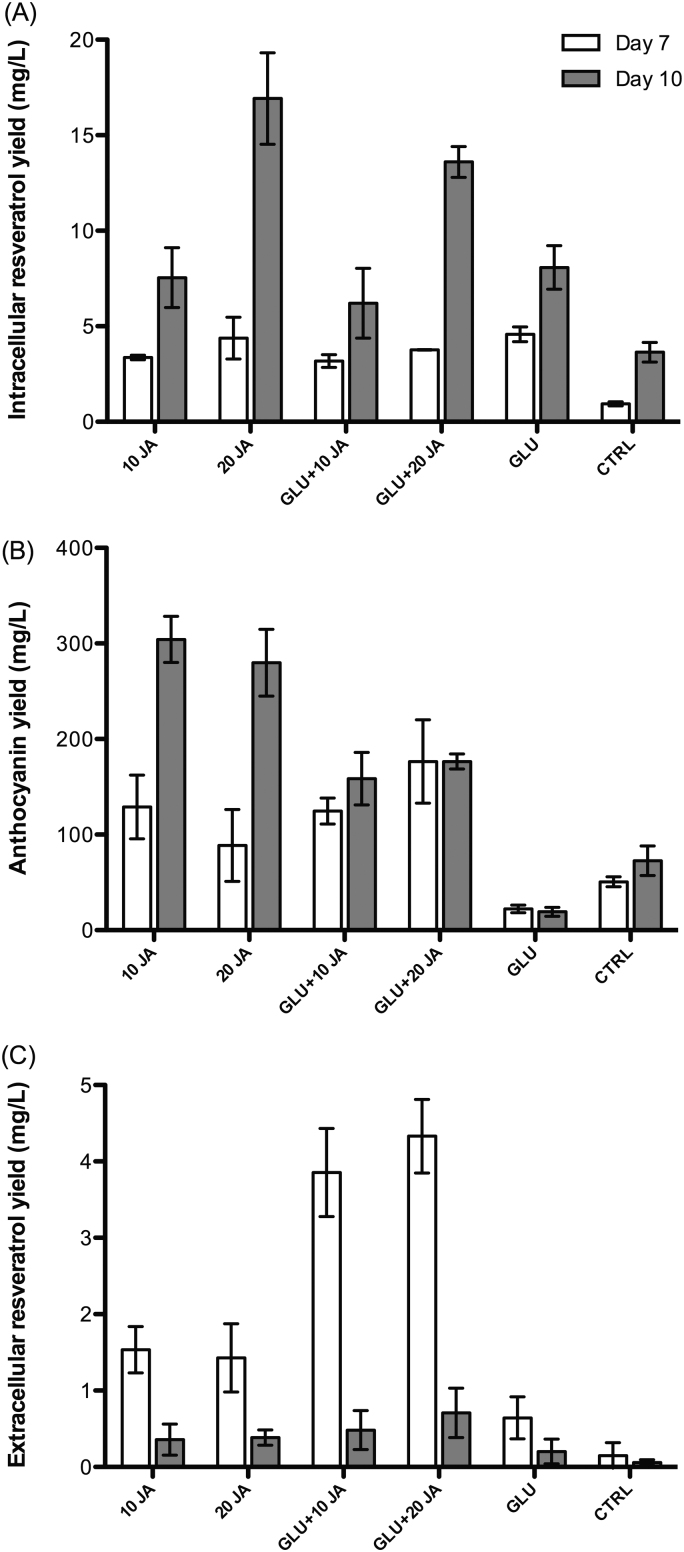
The production of intracellular resveratrol (A), anthocyanin (B), and extracellular resveratrol (C) in *V. vinifera* L. cell suspension cultures in response to the treatment with 10 μM, 20 μM JA, and in combination with 1 mg/L GLU; CTRL: control.

**Fig. 2:**
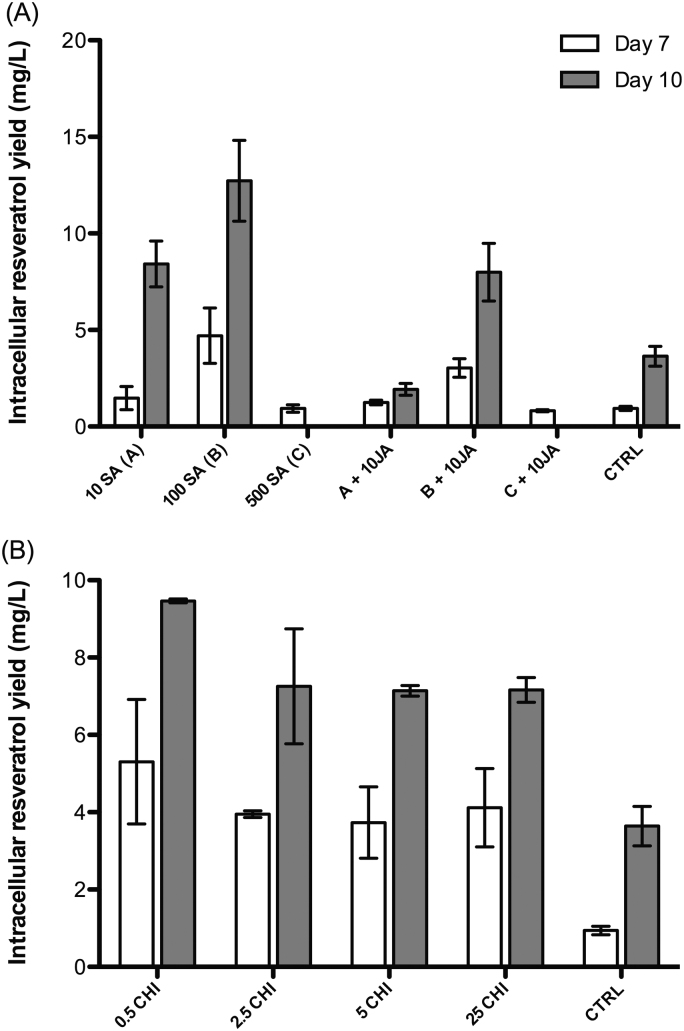
The production of intracellular resveratrol in *V. vinifera* L. cell suspension cultures in response to the treatment with 10 μM, 100 μM, and 500 μM SA and their combinations with 10 μM JA (A); and in response to the treatment with 0.5 mg/L, 2.5 mg/L, 5 mg/L and 25 mg/L CHI (B); CTRL: control.

JA was the only elicitor in this study that increased the production of both intracellular resveratrol (Fig. 1A) and anthocyanins (Fig. 1B). Curtin et al. [22] also reported the enhancement of anthocyanin biosynthesis in *V. vinifera* L. cell suspension cultures by JA and in combination with light irradiation. JA might activate the phenylpropanoid pathway, which provide substrates for both anthocyanin and resveratrol syntheses. As a result, total phenolics yield was increased several fold by the JA treatment (Table 1). The addition of JA was found to initiate the *de novo* transcription of genes responsible for the production of enzymes in the phenylpropanoid pathway [23]. SA at concentrations of 10 μM and 100 μM at least doubled the production of intracellular resveratrol at day 10 (Fig. 2A). However, when SA was combined with JA, a negative effect was observed. SA was previously proposed to inhibit the synthesis and signal transduction of JA [24].

The addition of CHI – a derivative of chitin – increased the level of intracellular resveratrol by around fivefold at day 7 (Fig. 2B). However, the difference in the level of intracellular resveratrol between the elicited cultures and the control was smaller at day 10. At much higher concentrations, CHI was also found to increase the intracellular accumulation of resveratrol from 3 to 10.5-fold in *V. vinifera* cv. Barbera cell cultures [25].

**Fig. 3:**
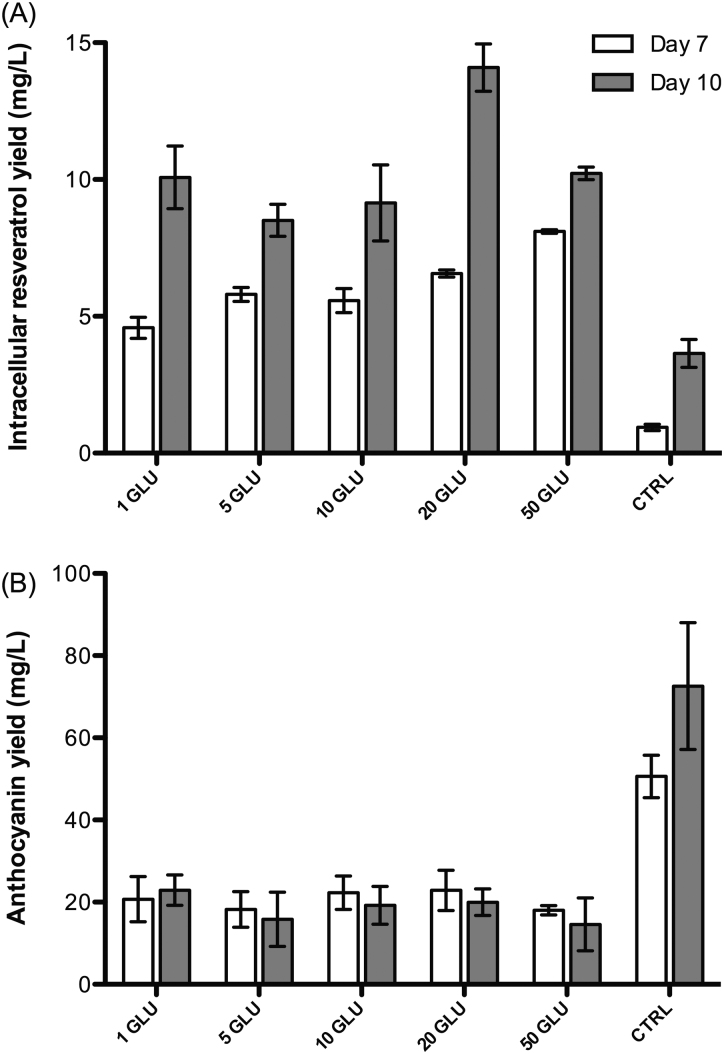
The production of intracellular resveratrol (A) and anthocyanin (B) in *V. vinifera* L. cell suspension cultures in response to the treatment with 1 mg/L, 5 mg/L, 10 mg/L, 20 mg/L and 50 mg/L GLU; CTRL: control.

Both chitin and glucan are major structural components of many fungi, and they are known to increase the accumulation of soluble pathogenesis-related proteins in plants [26]. Therefore, as is the case with CHI, the treatment with GLU at all tested concentrations increased the level of intracellular resveratrol by 5–7-fold at day 7 when compared with the control (Fig. 3A). Different from JA effects, GLU treatment lowered the production of anthocyanins (Fig. 3B). Stilbene synthase and chalcone synthase – the branch-point enzymes of the biosynthetic pathways of stilbenes and anthocyanins – are known to use the same substrates [1]. Therefore, the decrease in the level of anthocyanins is probably due to the competition for common substrates between these synthases. This type of relationship was also reported in pines, where pinosylvin – the product of stilbene synthase in *Pinus densiflora* – effectively inhibited the activities of chalcone synthase [27].

Among tested elicitors, only four: GLU, JA, SA and CHI, significantly increased the production of intracellular resveratrol several fold. However, the highest concentration of intracellular resveratrol in single or combined treatments of these elicitors was still low, ranging from 10 mg/L to 15 mg/L.

### 3.3 Production of extracellular phenolics and extracellular resveratrol

GLU, the combination of GLU and JA, as well as a particular combination between 100 μM SA and JA greatly increased the secretion of extracellular phenolics while they did not decrease the intracellular phenolic yield (Table 1). Therefore, the effect of GLU and its JA combinations on the production of extracellular resveratrol was investigated, as a proof of concept. The combined treatment with GLU and JA showed an additive effect as it increased the level of extracellular resveratrol to around 4 mg/L, which is approximately 2.5-fold higher that of JA single treatment (Fig. 1C). As GLU and JA increased the concentration of both intracellular and extracellular resveratrol, these elicitors probably affect the biosynthesis as well as the secretion of this stilbene.

It is noted that the level of extracellular resveratrol was always lower than that of intracellular resveratrol. Resveratrol is either preferentially not secreted into the medium or secreted but rapidly degraded by extracellular enzymes. The level of extracellular resveratrol may not be a true reflection of the amount of resveratrol secreted into the medium, as an indeterminate amount is probably being degraded. Therefore, it is crucial to have adsorbents in the medium to adsorb and store secreted resveratrol. The co-culture of Amberlite XAD-7 with cells, as discussed below, fulfills this requirement.

### 3.4 Resveratrol production by XAD-7 co-culture and elicitation

**Fig. 4:**
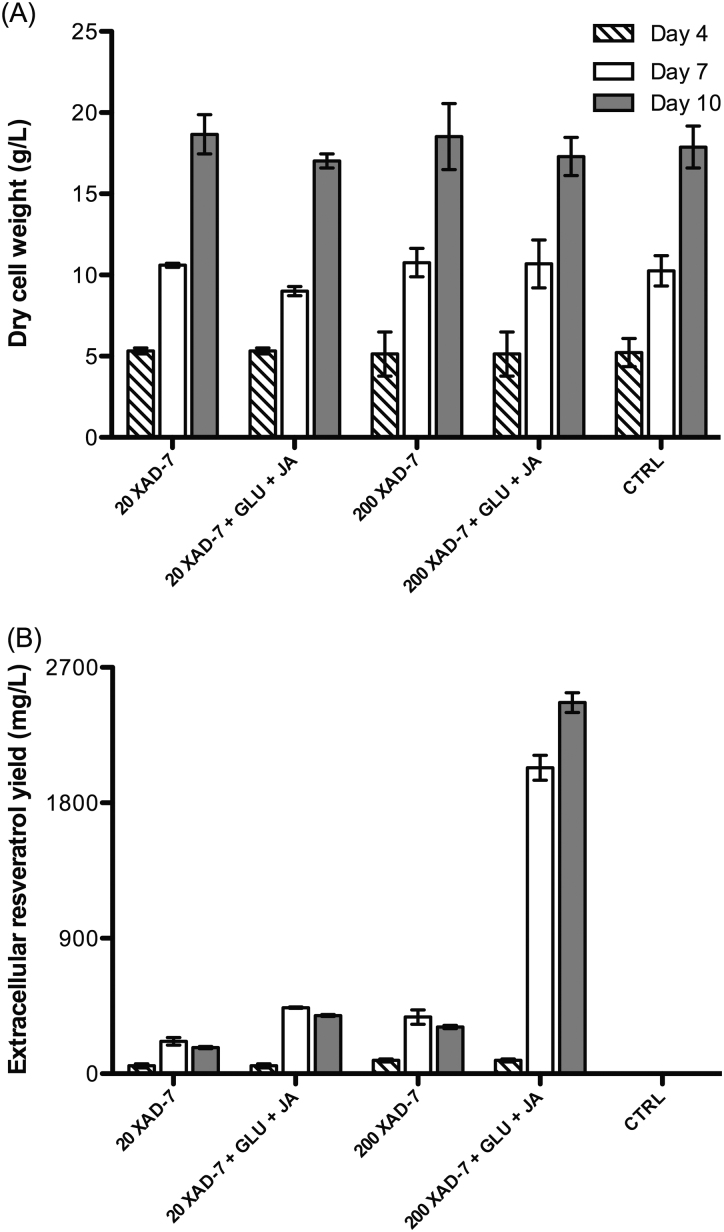
The dry cell weight (A) and the production of extracellular resveratrol (B) of *V. vinifera* L. cell suspension cultures co-cultured with 20 g/L or 200 g/L XAD-7, and in response to their combined treatments with 10 μM JA and 1 mg/L GLU; CTRL: control.

The co-culture with XAD-7, even at 200 g/L, did not cause any effect on cell growth (Fig. 4A). Interestingly, the combined treatment of JA and GLU, and the addition of XAD-7 resulted in a synergistic enhancement of resveratrol production in *V. vinifera* L. cell suspension cultures. In the combined treatment of 200 g/L XAD-7, 1 mg/L GLU and 10 μM JA, the total yields of resveratrol extracted from the XAD-7 beads were approximately 2100 mg/L at day 7 and 2400 mg/L at day 10 (Fig. 4B). In contrast, the level of extracellular resveratrol in the control was extremely low, which was 0.15 mg/L at day 7 and 0.06 mg/L at day 10. Therefore, the combined treatment of these two elicitors with XAD-7 increased the production of extracellular resveratrol by up to four orders of magnitude. The increased production of resveratrol worked in a XAD-7 dose-dependent manner. Approximately 215 mg/L resveratrol was extracted from beads of cultures with 20 g/L XAD-7 at day 7 while that from cultures with 200 g/L XAD-7 was about 376 mg/L. It is noted that the majority of secreted resveratrol was absorbed by XAD-7. The level of unbound resveratrol left in the medium was less than 0.8 mg/L. The level of resveratrol extracted from XAD-7 co-cultured with elicited cultures was approximately 2- to 8-fold higher than from XAD-7 in non-elicited cultures (Fig. 4B), indicating that the elicitors were still active in the presence of XAD-7.

Recently, a number of studies using grape cell suspension cultures have reported high yields of resveratrol. By using methyl jasmonate (MeJA, 200 μM) as the elicitor, Donnez et al. [10] produced up to 150 mg/L of resveratrol in a flask system and 209 mg/L in a 2 L-stirred bioreactor in *V. vinifera* cv. Chasselas × *V. berlandieri* cell suspension cultures. The addition of dimethyl-β-cyclodextrin (DIMEB) to *V. vinifera* cv. Gamay cultures produced a resveratrol yield of 100 mg/L [28]. Moreover, when this elicitor was added to *V. vinifera* cv. Monastrell albino cell suspension cultures, the yield was boosted up to 680 mg/L [16], and the combination between DIMED and MeJA produced an even higher concentration of resveratrol 8, 16. However, it is worth noting that high concentrations of elicitors (50 mM DIMED and 100 μM MeJA) were used, and the combination treatment caused a significant reduction in cell growth, by approximately 30% at day 5 of elicitation [16]. More importantly, the usage of XAD-7 in this current study would facilitate the purification of resveratrol in the downstream process as resveratrol-containing XAD-7 beads can be easily separated from cells, media and elicitors.

According to Collin and Edwards [12], a minimum yield of a desired product should be at least 2% of the total DCW for a fermenter system to become economic. The new culture process, combining elicitation and XAD-7 adsorption, meets this requirement. The average DCW of elicited or non-elicited cultures with or without XAD-7 is around 17 g/L (Fig. 4A) and the level of resveratrol produced after 6 days of treatment is 2400 mg/L (Fig. 4B). Therefore, the yield of resveratrol is approximately 14% of total DCW. This yield is relatively high compared with the yields of other compounds produced commercially by plant cell culture systems [12]. To facilitate the removal of XAD-7 and to ensure continuous fermentation processes in large-scale culture systems, the cells and XAD-7 beads can be separated by a semi-permeable membrane or the cells can be immobilized.

### 3.5 Relationship of resveratrol synthesis and degradation

Resveratrol, like other phytoalexins, is thought to be a transient constituent [29]; therefore, its accumulation is a reflection of both synthesis and degradation. The combined treatment of XAD-7, JA and GLU probably affects both processes. While the elicitors might induce the biosynthesis and secretion of resveratrol, XAD-7 probably acts as a safe, artificial storage site, preventing resveratrol from being degraded and derivatized. In the absence of XAD-7, the extracellular resveratrol level at day 10 was always lower than that at day 7 (Fig. 1C), although the level of intracellular resveratrol at day 10 was higher than that at day 7 (Fig. 1A). The degradation of extracellular resveratrol could be due to the activities of extracellular acidic peroxidases that were reported to degrade extracellular phytoalexins [30]. The appearance of extracellular ɛ-viniferin, which was tentatively identified based on its UV spectrum and HPLC retention time (Supplemental Fig. 1), supported the occurrence of peroxidative processes in the medium. The pattern for the production of this stilbene is identical to that of resveratrol, but its concentration is always lower than the level of resveratrol in the same experimental condition. The ratio of resveratrol to ɛ-viniferin levels in response to the combined treatment with 1 mg/L GLU and 10 μM JA is about 2–3-fold. In the presence of XAD-7, this ratio increased by several hundred-fold. This difference suggested that the adsorption by XAD-7 prevented resveratrol from its extracellular conversion.

### 3.6 Redirection for resveratrol synthesis

**Fig. 5:**
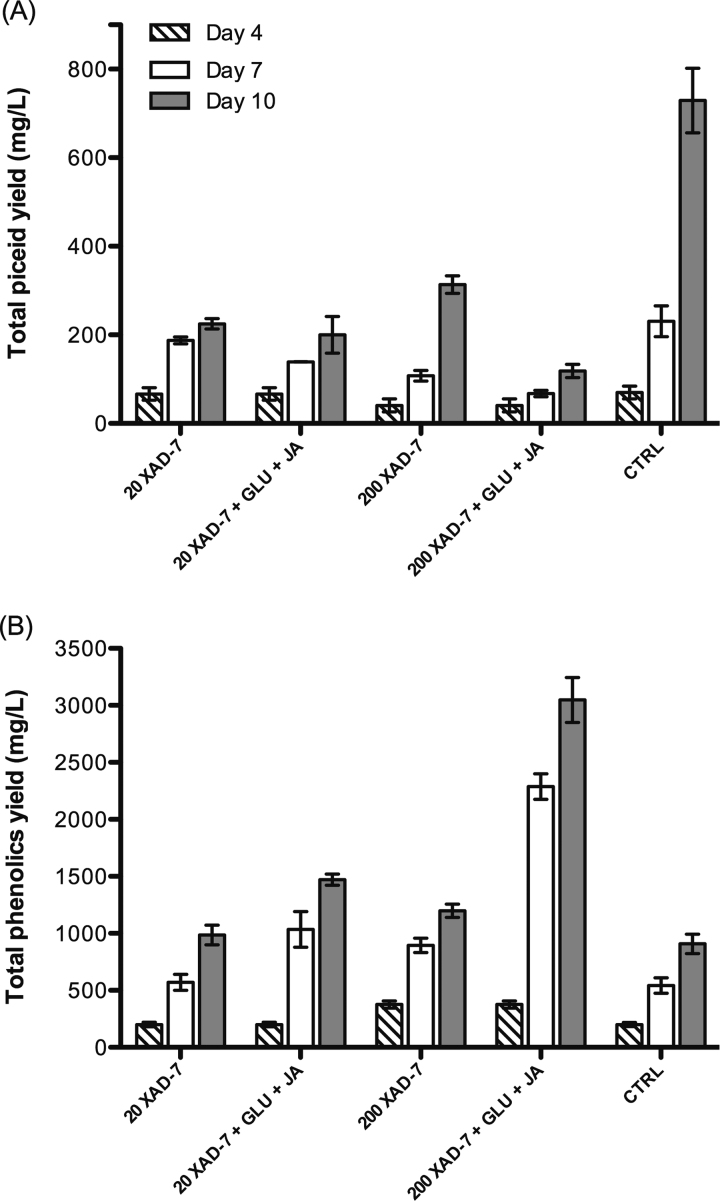
Total piceid (A) and total phenolics (B) extracted from the mixture of *V. vinifera* L. cells and XAD-7 beads (20 g/L and 200 g/L) in response to the combined treatment with 10 μM JA and 1 mg/L GLU; CTRL: control; gallic acid was used as the standard for the total phenolic determination.

Of stilbenes that were produced intracellularly, piceid was the most abundant (Supplemental Fig. 1). The average level of piceid at day 10 in controls was approximately 500 mg/L while that of intracellular resveratrol was less than 5 mg/L. However, when XAD-7 was added and adsorbed extracellular resveratrol, it probably created a concentration gradient of resveratrol from cells to the medium. As a result, there would be less intracellular resveratrol to be converted into piceid. Therefore, the total piceid yield was significantly reduced in response to the combined treatment of XAD-7 and elicitors (Fig. 5A). The total concentration of piceid at day 10 in the control was approximately 729 mg/L; however, in cultures treated with 200 g/L XAD-7 that level was just around 313 mg/L, and it was reduced further in the presence of elicitors (Fig. 5A). It is worth noting that resveratrol is the main phenolic that was released. The total phenolics concentrations in elicited cultures, which were co-cultured with 200 g/L XAD-7 at day 7 and day 10 were approximately 2300 mg/L and 3000 mg/L (Fig. 5B), while the levels of extracellular resveratrol extracted from the beads were 2100 mg/L and 2400 mg/L, respectively. A decrease in the level of other phenolics, accompanied with an increase in that of extracellular resveratrol suggests that the common precursors are redirected toward resveratrol production at the expense of other competing pathways.

## 4 Conclusion

The combined elicitation with JA and GLU, integrated with the addition of XAD-7 for the *in situ* removal and artificial storage of resveratrol resulted in a synergistic effect on resveratrol production. The level of resveratrol in response to the combined treatment with 200 g/L XAD-7, 1 mg/L GLU and 10 μM JA was approximately 2400 mg/L, which meets the requirement for a commercial culture process.

## Figures and Tables

**Figure:**
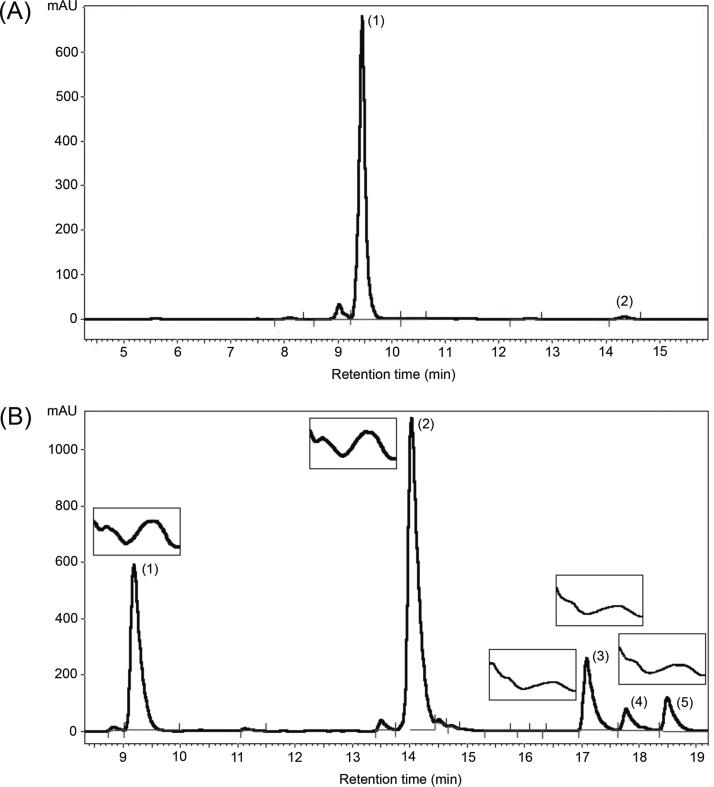


**Table 1:** The effects of betaine (BET), chitosan (CHI), β-glucan (GLU), methyl-β-cyclodextrin (MeCD), 3-methyl-salicyclic acid (MeSA), salicylic acid (SA), and jasmonic acid (JA) on dry cell weight, intracellular phenolics and extracellular phenolics productions of *V. vinifera* L. cell suspension cultures (*n* = 3, expressed as mean ± standard deviation).

Compound	Concentration	Dry cell weight (g/L)		Intracellular phenolics yield (mg/L)		Extracellular phenolics yield (mg/L)	
		Day 7	Day 10	Day 7	Day 10	Day 7	Day 10
BET	5 mM	10.1 ± 0.1	17.5 ± 0.9	328 ± 16	730 ± 80	40 ± 3	38 ± 4
	10 mM	10.7 ± 0.6	17.4 ± 0.3	350 ± 30	760 ± 80	35 ± 4	42 ± 4
	20 mM	9.9 ± 1.3	17.7 ± 0.6	330 ± 30	780 ± 50	34 ± 3	35 ± 3
	50 mM	11.7 ± 0.3	17.3 ± 0.2	410 ± 30	780 ± 60	29 ± 3	41 ± 3
	100 mM	11.1 ± 1.2	17.0 ± 0.2	360 ± 30	700 ± 60	36 ± 3	40 ± 5
	500 mM	12.1 ± 1.2	17.4 ± 0.2	386 ± 10	740 ± 50	29.0 ± 0.6	37 ± 4

CHI	0.5 mg/L	11.3 ± 0.9	18 ± 2	380 ± 20	760 ± 70	83 ± 9	51 ± 8
	2.5 mg/L	11.2 ± 1.4	18.3 ± 0.9	390 ± 30	720 ± 30	56 ± 8	44 ± 5
	5 mg/L	12 ± 2	18.3 ± 0.9	410 ± 60	700 ± 80	55 ± 8	52 ± 7
	25 mg/L	10.5 ± 1.3	17.3 ± 0.5	370 ± 40	605 ± 15	81 ± 8	60 ± 6

GLU	1 mg/L	9.7 ± 1.1	14.7 ± 0.3	350 ± 40	730 ± 40	95 ± 9	112 ± 10
	5 mg/L	8 ± 2	14.0 ± 0.2	359 ± 12	630 ± 50	109 ± 9	113 ± 10
	10 mg/L	9.6 ± 1.5	13.9 ± 1.5	390 ± 40	620 ± 60	101 ± 6	115.0 ± 1.7
	20 mg/L	9.5 ± 0.5	14.1 ± 0.4	370 ± 40	610 ± 60	102 ± 4	106.8 ± 1.6
	50 mg/L	9.7 ± 1.2	14.3 ± 0.5	370 ± 40	660 ± 30	102 ± 10	110 ± 9

MeCD	5 mg/L	9.6 ± 1.3	18.0 ± 0.4	379 ± 18	850 ± 90	31.1 ± 1.4	36 ± 3
	10 mg/L	10.1 ± 0.3	18.2 ± 0.4	380 ± 30	850 ± 60	45 ± 6	47 ± 6
	20 mg/L	9.9 ± 1.8	18.7 ± 0.2	398 ± 14	830 ± 50	33 ± 5	41 ± 5
	50 mg/L	10.2 ± 1.3	18.8 ± 0.5	390 ± 30	820 ± 70	41 ± 6	44 ± 6

MeSA	5 μM	11.9 ± 1.3	17.8 ± 1.9	391 ± 10	780 ± 80	24 ± 3	42 ± 5
	10 μM	13.4 ± 0.7	17.0 ± 0.2	417 ± 17	780 ± 70	23.0 ± 1.0	41 ± 4
	20 μM	12.0 ± 0.8	16.8 ± 0.1	405 ± 5	760 ± 60	26 ± 3	33.6 ± 1.9
	50 μM	12.0 ± 0.6	16.4 ± 0.5	360 ± 40	790 ± 30	24 ± 2	39 ± 4
	100 μM	11.8 ± 1.4	16.7 ± 0.6	390 ± 40	760 ± 40	26.3 ± 1.7	36 ± 3
	300 μM	10.1 ± 1.4	14.0 ± 0.8	330 ± 30	487 ± 16	90 ± 9	107 ± 10
	600 μM	9.3 ± 0.6	12.7 ± 0.7	293 ± 18	430 ± 20	103 ± 11	124 ± 13

SA	10 μM	12.4 ± 0.8	18.5 ± 0.8	370 ± 20	600 ± 20	20 ± 3	44.0 ± 0.2
	100 μM	11.7 ± 1.5	18.5 ± 1.5	380 ± 30	560 ± 50	22 ± 4	42 ± 4
	500 μM	8.3 ± 0.9	12.2 ± 1.2	280 ± 30	310 ± 20	92 ± 10	114 ± 13

JA	10 μM	10.8 ± 0.4	16.6 ± 1.4	700 ± 80	1480 ± 80	49 ± 6	47 ± 6
	20 μM	10.6 ± 1.3	16.4 ± 1.3	570 ± 60	1470 ± 170	77 ± 7	46 ± 5
	10 μM + 1 mg/L GLU	9.4 ± 1.1	15.2 ± 1.4	630 ± 50	1180 ± 90	130 ± 11	109 ± 12
	20 μM + 1 mg/L GLU	11.2 ± 1.5	15.7 ± 1.9	700 ± 80	1200 ± 50	121 ± 17	138 ± 7
	10 μM + 10 μM SA	10.5 ± 0.8	17 ± 2	1050 ± 70	1780 ± 110	30 ± 4	54 ± 6
	10 μM + 100 μM SA	9.0 ± 1.2	11.6 ± 1.8	480 ± 40	1180 ± 120	113 ± 13	116 ± 13
	10 μM + 500 μM SA	5.2 ± 0.7	5.8 ± 0.3	229 ± 17	210 ± 30	252 ± 6	214 ± 12

Control		10.9 ± 0.8	17.2 ± 0.9	380 ± 30	760 ± 60	33 ± 4	41 ± 4
